# Cage-bell Pt-Pd nanostructures with enhanced catalytic properties and superior methanol tolerance for oxygen reduction reaction

**DOI:** 10.1038/srep24600

**Published:** 2016-04-15

**Authors:** Dong Chen, Feng Ye, Hui Liu, Jun Yang

**Affiliations:** 1 State Key Laboratory of Multiphase Complex Systems, Institute of Process Engineering, Chinese Academy of Sciences, Beijing 100190, China; 2 University of Chinese Academy of Sciences, No. 19A Yuquan Road, Beijing 100049, China; 3 Center for Mesoscience, Institute of Process Engineering, Chinese Academy of Sciences, Beijing, 100190, China

## Abstract

Precisely tailoring the structure and fully making use of the components of nanoparticles are effective to enhance their catalytic performance for a given reaction. We herein demonstrate the design of cage-bell structured Pt-Pd nanoparticles, where a Pd shell is deliberately selected to enhance the catalytic property and methanol tolerance of Pt for oxygen reduction reaction. This strategy starts with the synthesis of core-shell Pt@Ag nanoparticles, followed by galvanic replacement reaction between the Ag shell and Pd^2+^ ions to form core-shell-shell Pt@Ag@Ag-Pd nanoparticles with a Pt core and double shells composed of Ag at inner and alloy Ag-Pd at outer, respectively. Then, the core-shell-shell templates are agitated with saturated NaCl solution to eliminate the Ag component from the double shells, leading to the formation of bimetallic Pt-Pd nanoparticles with a cage-bell structure, defined as a movable Pt core enclosed by a porous Pd shell, which show enhanced catalytic activity for oxygen reduction compared with that of the Pt seeds due to the additional catalysis from Pd shell. In addition, owing to the different diffusion behavior of methanol and oxygen molecules in the porous Pd shell, the Pt-Pd cage-bell nanostructures also exhibit superior methanol tolerant property in catalyzing the oxygen reduction.

Increasing the selectivity of Pt-based electrocatalysts for ORR is an effective way to overcome the methanol crossover from the anode to the cathode, one of the major problems in direct methanol fuel cells (DMFCs), which leads to a significant reduction of the fuel cell efficiency by creating a mixed potential at the cathode^1^,^2^,^3^. We have demonstrated the concept in our previous studies that a good ORR selectivity of the platinum (Pt) catalyst could be realized through a cage-bell structured (CBS) geometry^4^,^5^,^6^, or so-called yolk-shell structure in some reports^7^,^8^, which refers to a movable core enclosed by a porous shell. In the CBS nanoparticles, the catalytically active metal, i.e. Pt, was placed at the core region shielded by a porous metal shell, e.g. ruthenium (Ru), Osmium (Os), or iridium (Ir), which is inactive for methanol oxidation. Although the metal shells could effectively inhibit the methanol oxidation reaction (MOR) by preventing the methanol molecules from diffusing into the interior of CBS particles, the original design is not a cost-effective one. The metal shells (Ru, Os, or Ir) cause an extra cost of Pt electrocatalysts but do not have contribution to the ORR since they are also inactive for the oxygen reduction.

Therefore, in this work we aim at finding a more economic route to produce CBS nanoparticles with an ORR active Pt core and a deliberately selected metal shell, which is inert for MOR but active for ORR, and palladium (Pd) is among the candidates for the shell component. In our recent progress in synthesis of hollow structured Pd nanoparticles, we found that the galvanic replacement reaction between the silver (Ag) particles and Pd^2+^ ions would result in the formation of core-shell nanostructures with an Ag core and an Ag-Pd alloy shell (Ag@Ag-Pd)^9^,^10^,^11^,^12^, and this finding lays the foundations for the strategy developed in this work. In brief, core-shell Pt@Ag nanoparticles with a Pt core and an Ag shell are firstly prepared by reducing the Ag^+^ precursors in the presence of pre-synthesized Pt seed particles in oleylamine. Then galvanic replacement reaction between the Ag shell and Pd^2+^ ions is conducted for the formation of core-shell-shell Pt@Ag@Ag-Pd nanoparticles with a Pt core and double shells composed of Ag at inner and alloy Ag-Pd at the outer regions, respectively. Subsequently, the core-shell-shell Pt@Ag@Ag-Pd templates are agitated with NaCl solution to eliminate the Ag component from the inner and outer shells for the formation of final Pt-Pd nanoparticles with a cage-bell structure, defined as a movable Pt core enclosed by a porous Pd shell. As we will display later, the CBS Pt-Pd nanoparticles show superior activity, durability, and selectivity for the ORR in the presence of high concentration of methanol in comparison with those of the staring Pt seed particles. In addition, the concept in this work might be extended to generate other CBS nanoparticles with enhanced activity and desired selectivity for a given chemical reaction.

## Results and Discussion

**Figure 1:**
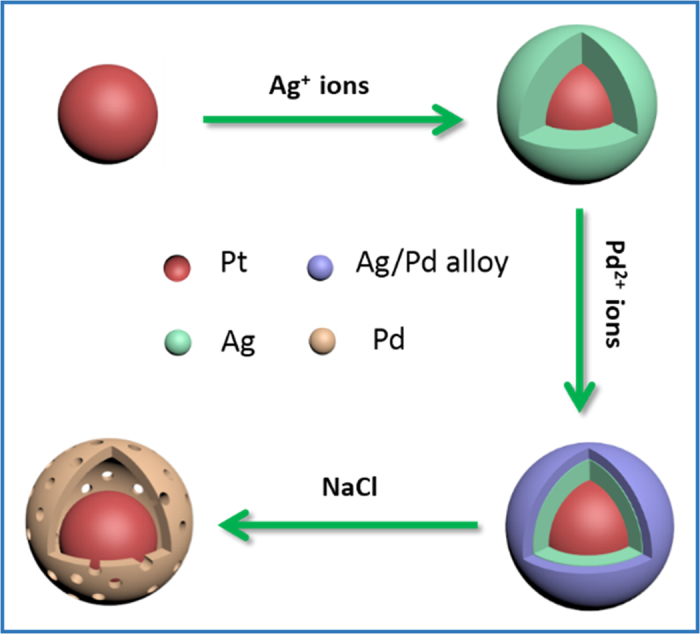
Synthetic strategy. Schematic illustration for the formation of cage-bell structured Pt-Pd nanoparticles using core-shell-shell Pt@Ag@Ag-Pd nanoparticles as templates, which are prepared by galvanic replacement between the Ag shell of core-shell Pt@Ag nanoparticles and Pd^2+^ ion precursors.

Figure 1 is the schematic illustration for the formation of bimetallic Pt-Pd nanoparticles with a cage-bell structure. Analogous to the synthesis of CBS Pt-M (M = Ru, Os, or Ir) nanoparticles we reported earlier^4^,^6^, the protocol in this study also begins with the preparation of Pt seed particles by oleylamine reduction of Pt(II) acetylacetonate (Pt(acac)_2_). As shown by Figure S1a of Supplementary Information (SI), the XRD pattern demonstrates the successful synthesis of face-centered cubic (fcc) Pt nanoparticles^13^. For the later comparison, the XRD patterns of monometallic Ag and Pd nanoparticles prepared in oleylamine were also presented, as shown by SI Figure S1b,c, respectively. The transmission electron microscope (TEM) image (SI Figure S2a) manifests that with the assistance of small amount of AgNO_3_, the obtained Pt seeds are dominated by spherical particles, which are nearly mono-dispersed and have an average diameter of ca. 5.9 nm. The high-resolution TEM (HRTEM) image (SI Figure S2b) illustrates the lattice planes in these nanoparticles, showing an interplanar spacing of ca. 0.23 nm, which corresponds to the (111) planes of fcc Pt (JCPDS Card File 882343).

### Core-shell Pt@Ag and core-shell-shell Pt@Ag@Ag-Pd nanoparticles

**Figure 2:**
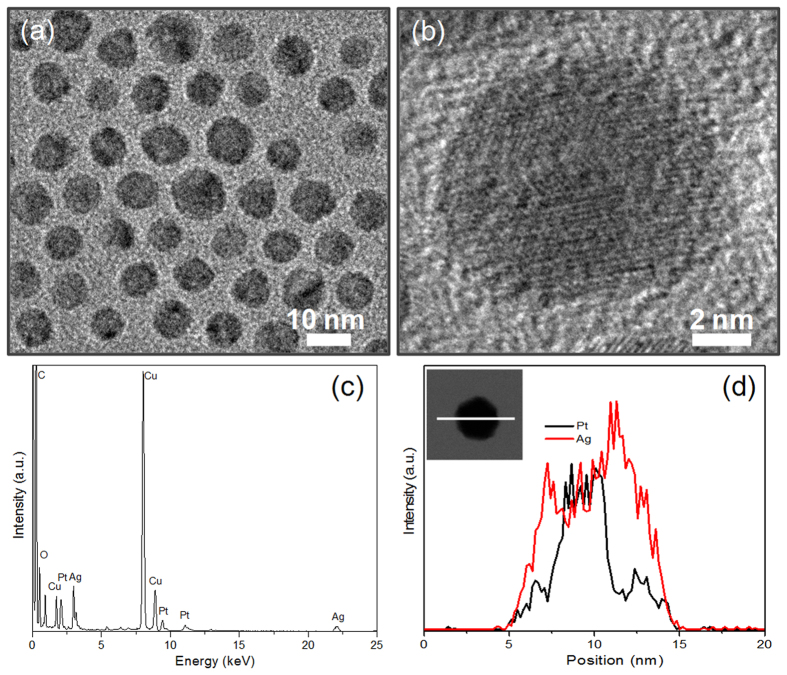
Core-shell Pt@Ag nanoparticles. TEM image (**a**), HRTEM image (**b**), STEM-EDX analysis (**c**), and elemental profiles (**d**) of the core-shell Pt@Ag nanoparticles as-prepared by seed-mediated growth method.

The Pt seed particles were then used to synthesize core-shell Pt@Ag nanoparticles *via* seed-mediated growth. The TEM and HRTEM images shown in Fig. 2a,b indicate that these well-dispersed particles possess a quasi-spherical shape with an average size of ca. 9.0 nm. The EDX analysis in scanning TEM (STEM) mode illustrates the particles formed *via* seed-mediated growth are indeed composed of Pt and Ag (Fig. 2c). The core-shell construction of these particles could be confirmed by the elemental profiles of a single particle in STEM mode. As shown in Fig. 2d, along the white line across the nanoparticle (the inset of Fig. 2d), the signal of Pt is confined to core region whereas the Ag signal is present throughout the particle. Compared with that of the Pt seeds, a slight shift to lower angles is noted in the XRD peaks for the core-shell Pt@Ag nanoparticles (SI Figure S1d), which is attributed to the smaller lattice parameter of Pt than that of Ag (0.392 nm for Pt and 0.409 nm for Ag, respectively)^14^. In addition, the core-shell Pt@Ag nanoparticles show a broad absorption band centered at 367.1 nm (SI Figure S3b), which could be attributed to the surface plasmon resonance (SPR) of Ag shell since Pt has no characteristic UV-visible absorption peak. The large blue-shift (ca. 40.2 nm) for the Ag surface plasmon band in the core-shell Pt@Ag nanoparticles relative to the Ag nanoparticles prepared by oleylamine reduction of AgNO_3_ (ca. 407.3 nm, SI Figure S3a) might be an indication to show the effect from Pt core on the optical property of Ag shell.

In the strategy developed in this work, the preparation of core-shell-shell Pt@Ag@Ag-Pd nanoparticles with an inner Ag shell and an alloy Ag-Pd outer shell is an important step proceeding the generation of CBS Pt-Pd nanoparticles with a movable Pt core enclosed by a porous Pd shell. The core-shell-shell Pt@Ag@Ag-Pd naoparticles were synthesized *via* galvanic replacement reaction between Pd^2+^ and the Ag shell of core-shell Pt@Ag nanoparticles, which could be described as 2Ag + Pd^2+^ → Pd + 2Ag^+^. The exterior of the pure Ag shells of core-shell Pt@Ag nanoparticles are transformed into a shell made of Ag-Pd alloy by galvanic replacement reaction, which inhibits the further oxidative dissolution of the interior of the Ag shells^9^,^11^,^15^, leading to the formation of double shells composed of Ag at inner and alloy Ag-Pd at outer, respectively, on the Pt cores. Actually, the real process occurred in the solution is more complicated. In addition to the replacement reaction between Ag shells and Pd^2+^ precursors, it also involves the oleylamine reduction of Ag^+^ ions generated from galvanic replacement, the reduction of Pd^2+^ ions added to the solution, and the alloying between Ag and Pd atoms. Overall, these closely knitted reactions collectively results in the products of core-shell-shell Pt@Ag@Ag-Pd nanoparticles with an Ag layer at inner and an alloy Ag-Pd layer at outer instead of core-shell Pt@Ag-Pd nanoparticles with a complete alloy Ag-Pd shell.

**Figure 3:**
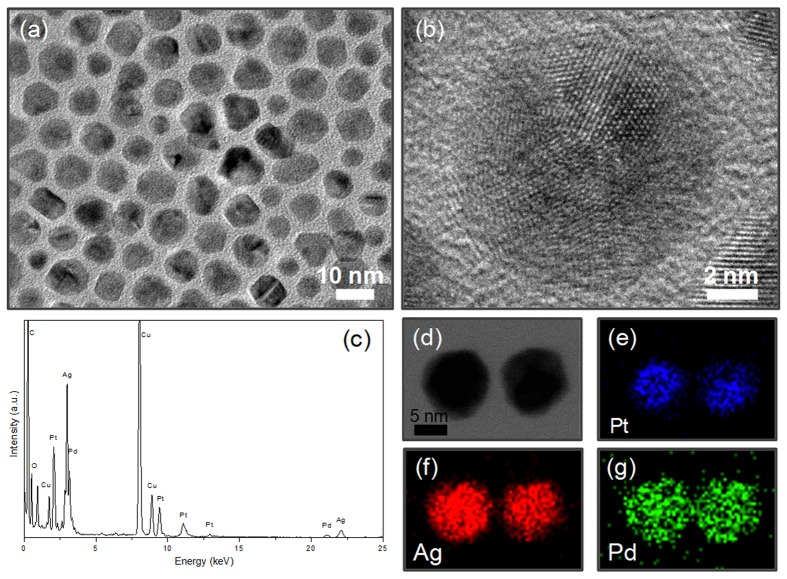
Core-shell-shell Pt@Ag@Ag-Pd nanoparticles. TEM image (**a**), HRTEM image (**b**), STEM-EDX analysis (**c**), STEM image (**d**), and elemental mappings (**e–g**) of core-shell-shell Pt@Ag@Ag-Pd nanoparticles as-prepared by galvanic replacement reaction between the Ag shell of Pt@Ag nanoparticles and Pd^2+^ ions in oleylamine.

The TEM and HRTEM images displayed in Fig. 3a,b show the formed core-shell-shell Pt@Ag@Ag-Pd nanoparticles *via* the replacement reaction between Pd^2+^ ions and the Ag shells of core-shell Pt@Ag nanoparticles, which maintain the quasi-spherical morphology with an average diameter of 8.9 nm. The STEM-EDX analysis (Fig. 3c) of two arbitrarily selected particles indicated in Fig. 3d proves that the products are indeed composed of Pt, Ag and Pd, and their core-shell-shell structure could be verified by the elemental mappings (Fig. 3e–g) of the two particles in Fig. 3d, which reveal that Pt is mainly centered in the core region (Fig. 3e), while the Ag and Pd components are distributed throughout the entire particle (Fig. 3f for Ag and Fig. 3g for Pd, respectively). It was noteworthy that the XRD pattern of core-shell-shell Pt@Ag@Ag-Pd nanoparticles (SI Figure S1e) is quite analogous to that of core-shell Pt@Ag nanoparticles (SI Figure S1d) due to the presence of limited Pd amount in the outer shell. However, as shown by SI Figure S3b,c, respectively, a perceivable blue-shift (ca. 26.7 nm) of the adsorption band is observed for the core-shell-shell Pt@Ag@Ag-Pd nanoparticles in comparison with that of the core-shell Pt@Ag nanoparticles, implying the variation in the chemical composition of the Ag shell *via* galvanic replacement reaction^16^.

### Bimetallic Pt-Pd nanoparticles with cage-bell structure

**Figure 4:**
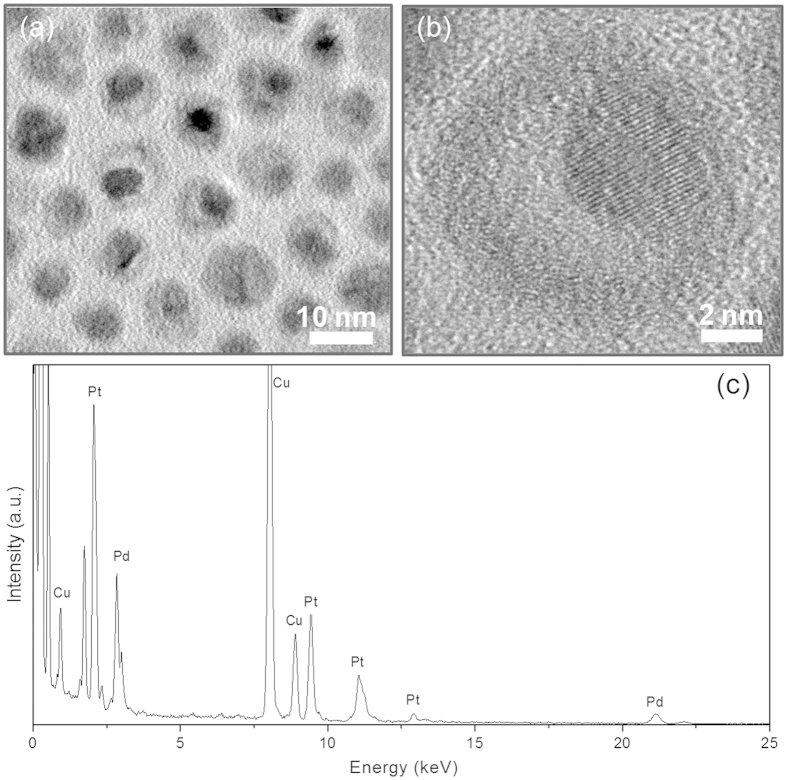
Cage-bell structured Pt-Pd nanoparticles. TEM image (**a**), HRTEM image (**b**), and EDX analysis (**c**) of the cage-bell structured Pt-Pd nanoparticles as-prepared by removing the Ag component from the inner and outer shells of core-shell-shell Pt@Ag@Ag-Pd nanoparticles with saturated NaCl solution.

As has been confirmed in a number of literatures, the Ag component is easily etched by Cl^−^ anions and dissolved O_2_ in solution^4^,^17^,^18^,^19^,^20^. Therefore, after aging the mixture of core-shell-shell Pt@Ag@Ag-Pd colloidal solution in toluene and saturated aqueous NaCl solution under vigorous stirring, the Ag component in the outer alloy Ag-Pd shells of the core-shell-shell Pt@Ag@Ag-Pd nanoparticles could be firstly etched into Ag^+^ cations, which subsequently react with Cl^−^ anions to form AgCl dissolved in the saturated NaCl solution. The elimination of Ag from the outer alloy Ag-Pd shells results in the establishment of the ionic channel for further removal of Ag from the inner shell region of the core-shell-shell Pt@Ag@Ag-Pd nanoparticles, finally leading to the formation of bimetallic Pt-Pd nanoparticles with a cage-bell structure. After treatment with NaCl, the ICP-OES (inductively coupled plasma-optical emission spectrometry) does not detect the Ag content, verifying the successful removal of Ag from the core-shell-shell Pt@Ag@Ag-Pd nanoparticles, and this is in accord with the disappearance of the Ag signal in the EDX spectrum (Fig. 4c), and the obliteration of the adsorption band of Ag in the UV-Vis spectrum for the final bimetallic Pt-Pd products (SI Figure S3d). The TEM and HRTEM images of the core-shell-shell Pt@Ag@Ag-Pd particles after NaCl treatment were shown in Fig. 4a,b, respectively. As indicated, the void space between the Pt core and the outer Pd shell regions, formed upon the elimination of the inner Ag shell by NaCl, is clearly discernible by the strong brightness contrast. The formation of CBS Pt-Pd nanoparticles could conversely prove that the Ag shells in core-shell Pt@Ag nanoparticles are not completely transformed into alloy Ag-Pd shells but into double shells with Ag at inner and alloy Ag-Pd at outer region, respectively, during the galvanic replacement reaction process. In addition, the electron microscopy images manifest that the size and morphology of the core-shell-shell templates are preserved in the cage-bell structured products. Notably, the XRD pattern of CBS Pt-Pd nanoparticles (SI Figure S1f) has a positive shift towards high angles compared with that of their core-shell-shell templates (SI Figure S1e) because of the removal of Ag component, which has larger lattice parameters.

### Electrochemical properties of Pt seeds and CBS Pt-Pd nanoparticles

To evaluate the electrochemical properties of CBS Pt-Pd nanoparticles for ORR and their methanol tolerant property at room temperature, both the CBS Pt-Pd nanoparticles and Pt seed nanoparticles were loaded on Vulcan carbon substrates with mass ratio of 20% (Pt base), labeled as CBS Pt-Pd/C and Pt/C respectively. The carbon monoxide (CO) stripping voltammograms of the Pt/C and CBS Pt-Pd/C shown in SI Figure S4 were used to determine the electrochemically active surface areas (ECSAs) of the corresponding catalysts^21^. The ECSAs normalized by the mass of Pt are 60.3 m^2^ g^−1^ and 79.5 m^2^ g^−1^ for Pt/C and CBS Pt-Pd/C, respectively. The larger ECSAs of the CBS Pt-Pd catalysts should be attributed to the adsorption of CO on both the Pt cores and Pd shells in CBS particles.

**Figure 5:**
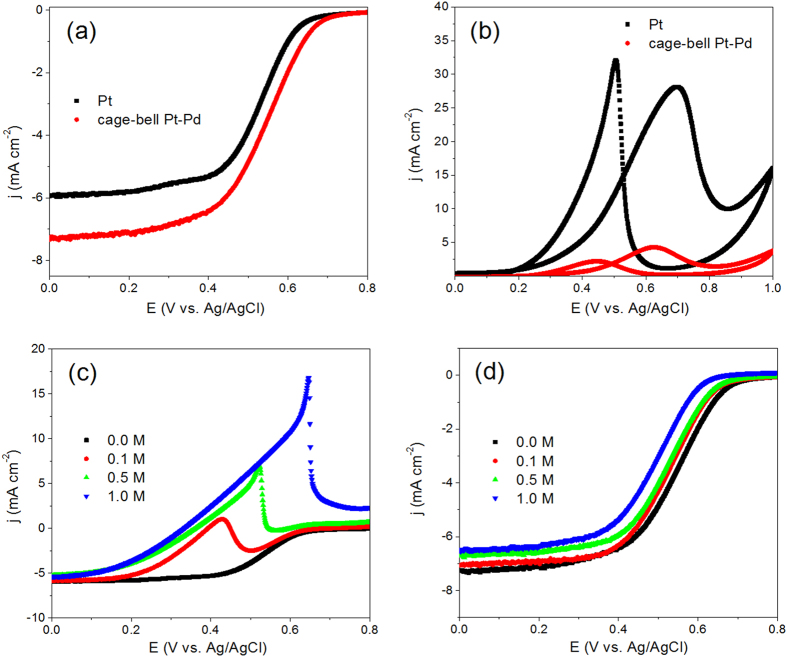
Electrochemical measurements. ORR polarization curves of Pt/C and CBS Pt-Pd/C catalysts in an O_2_-saturated HClO_4_ solution (0.1 M) at 10 mV s^−1^ and a rotating speed of 1600 rpm (**a**); cyclic voltammograms of Pt/C and CBS Pt-Pd/C catalysts in nitrogen-purged HClO_4_ (0.1 M) with 1 M methanol at 20 mV s^−1^ (**b**); ORR polarization curves of Pt/C (**c**) and CBS Pt-Pd/C (**d**) catalysts in an O_2_-saturated HClO_4_ solution (0.1 M) with 0.0 M, 0.1 M, 0.5 M and 1.0 M methanol, respectively, at a scan rate of 10 mV s^−1^ and a rotating speed of 1600 rpm.

The ORR polarization curves of Pt/C and CBS Pt-Pd/C were shown in Fig. 5a. Apparently, compared with the Pt/C catalyst, the CBS Pt-Pd/C has a more positive half-wave potential for ORR (523 mV for Pt/C and 547 mV for CBS Pt-Pd/C, respectively). The current density of CBS Pt-Pd/C at 0.2 V (7.11 mA cm^−2^) is 1.2 times as that of Pt/C (5.86 mA cm^−2^), clearly illustrating the CBS Pt-Pd/C has higher activity than that of Pt/C for ORR. Considering the same size and loading of Pt on the carbon substrates, the enhanced catalytic activity of CBS Pt-Pd nanoparticles for ORR could be attributed to the additional catalysis induced by the porous Pd shell, which is active for ORR. In contrast, the voltammograms of the MOR for Pt/C and CBS Pt-Pd/C displayed in Fig. 5b are quite different. The peak current density for CBS Pt-Pd/C associated with methanol oxidation in the forward scan is 4.3 mA cm^−2^ and is only 15.2% of the current density of Pt/C (28.2 mA cm^−2^), indicating that CBS Pt-Pd particles have much lower catalytic activity for MOR than that of Pt seeds. It has been well known that Pd is an inert metal for MOR in acidic solutions^22^,^23^, and hence the poor activity of CBS Pt-Pd/C for MOR could be ascribed to the presence of porous Pd shells, which prevent methanol molecules from accessing the Pt core by inhibiting their diffusion through the porous shells.

To further verify the methanol tolerant property of CBS Pt-Pd nanoparticles for ORR, the catalysis of Pt/C and CBS Pt-Pd/C for ORR were carried out in 0.1 M HClO_4_ containing 0.0, 0.1, 0.5, and 1.0 M methanol, respectively. As shown by Fig. 5c, in the presence of methanol, the ORR polarization curves for Pt/C catalysts have been seriously influenced, and the peaks at the potential for methanol oxidation are clearly observed. With the increase of methanol concentration in electrolyte, the peak current density associated with the methanol oxidation increases sharply, indicating quite poor methanol tolerance of Pt/C for ORR. However, the ORR polarization curves for CBS Pt-Pd/C are hardly affected by methanol, as exhibited by Fig. 5d. Specifically, with the methanol concentration of 0.1 M, 0.5 M and 1 M in the electrolyte, the half-wave potential of CBS Pt-Pd/C catalysts can reach 96.9%, 94.8% and 89.8% of half-wave potential of the same CBS particles for ORR in the absence of methanol, implying their superior methanol tolerance. Analogous to the CBS particles we depicted in early studies^4^,^6^, for the CBS Pt-Pd catalyst, the methanol or oxygen also must diffuse through the porous Pd shell to access the active Pt core for the occurrence of the electrocatalytic reaction. In this case, the larger molecular size of methanol would limit its diffusion in the porous shell of CBS particles, rendering the oxidation of methanol a non-competitive event. Chronoamperometries of Pt/C and CBS Pt-Pd/C catalysts at 0.55 V in oxygen-saturated 0.1 M HClO_4_ solution with 0.5 M methanol were used to obtain some indications of the long-term performance of the catalysts for ORR. SI Figure S5 shows that the “steady state” activity of CBS Pt-Pd nanoparticles is much higher than that of the Pt seeds after more than 2 h, indicating that the durability of Pt catalyst for ORR in the presence of methanol can be enhanced by the porous Pd shell.

## Conclusions

In summary, we have developed an economic approach to produce bimetallic Pt-Pd nanoparticles with a cage-bell structure, which is based on the removal of the inner Ag shells from the core-shell-shell Pt@Ag@Ag-Pd nanoparticles with a Pt core and double shells composed of an inner Ag shell and an outer alloy Ag-Pd shell, respectively, using saturated NaCl solution. The electrochemical measurments demonstrated that the NaCl induced CBS Pt-Pd nanoparticles supported on carbon support exhibit enhanced ORR activity and superior methanol tolerance due to the deliberately selected Pd shells, which contribute additional catalysis for oxygen reduction and stop methanol accessing the Pt core. In particular, the design for sufficiently making use of various components in a nanostructure might be extended to generate other heterogeneous materials with enhanced activity and desired selectivity for a given chemical reaction.

## Methods

### General chemicals

Silver nitrate (AgNO_3_, ACS reagent, ≥99.0%), platinum(II) acetylacetonate (Pt(acac)_2_, 97%), palladium(II) acetylacetonate (Pd(acac)_2_, 99%), oleylamine (70%, technical grade) and Nafion 117 solution (5% in a mixture of lower aliphatic alcohols and water) were purchased from Sigma-Aldrich. Ethanol (>99.7%), methanol (>99%), toluene (>99.5%), perchloric acid solution (70%), sodium chloride (NaCl, analytical grade) and acetic acid (C_2_H_4_O_2_, analytical grade) were purchased from Beijing Chemical Works. Vulcan XC-72 carbon powders (XC-72C with BET surface area of 250 m^2^ g^−1^ and average particle size of 40 ∼ 50 nm) were purchased from Cabot. All chemicals were used as received. Deionized water was distilled by a Milli-Q Ultrapure-water purification system. All glassware and Teflon-coated magnetic stirring bars were cleaned with *aqua regia*, followed by copious rinsing with deionized water before drying in an oven.

### Synthesis of core-shell Pt@Ag nanoparticles

Briefly, 40 mg of Pt(acac)_2_ and 3 mg of AgNO_3_ were dissolved in 10 mL of oleylamine placed in a three-necked flask equipped with a condenser and stir bar. The small amount of AgNO_3_ was used to facilitate the formation of Pt nanoparticles with regularly spherical morphologies. The solution was heated to 170 °C and kept at this condition for 3 h for the complete reduction of Pt^2+^ ions by oleylamine, which also serves as the capping agent. Then, the temperature was cooled down to 150 °C, and 17 mg of AgNO_3_ was swiftly added under vigorous stirring. The mixture was kept at this temperature for 2 h, resulting in the formation of core-shell Pt@Ag colloidal solution. After the reaction, the core-shell Pt@Ag nanoparticles were purified by precipitation with methanol, followed by centrifugation and washing with methanol, then re-dispersed in 10 mL of toluene.

### Synthesis of core-shell-shell Pt@Ag@Ag-Pd nanoparticles

Galvanic replacement reaction was used to synthesize core-shell-shell Pt@Ag@Ag-Pd nanoparticles with a Pt core and double shells composed of Ag and alloy Ag-Pd at inner and outer regions, respectively. In detail, to the core-shell Pt@Ag colloidal solution in oleylamine at 150 °C, 9 mg of Pd(acac)_2_ was swiftly introduced. The mixture was kept at this temperature for 2 h to fulfill the replacement reaction between Ag shells and Pd^2+^ ions for the formation of core-shell-shell Pt@Ag@Ag-Pd nanoparticles, which were recovered by precipitation with methanol, followed by centrifugation and washing with methanol, and then re-dispersed in 10 mL toluene.

### Preparation of CBS Pt-Pd nanoparticles

For the preparation of CBS Pt-Pd nanoparticles with a movable Pt core enclosed by a porous Pd shell, the core-shell-shell Pt@Ag@Ag-Pd nanoparticles dispersed in toluene were mixed with 20 mL of saturated aqueous NaCl solution, and the mixture was aged for 48 h under vigorous stirring at room temperature for the complete removal of Ag component from the inner Ag and outer Ag-Pd alloy shells. Afterwards, the upper toluene phase containing CBS Pt-Pd nanoparticles was collected after complete separation of two phases.

### Particle characterizations

Transmission electron microscopy (TEM), high-resolution TEM (HRTEM), and scanning TEM (STEM) were performed on a JEOL JEM-2010F electron microscope operated at 200 kV with the supplied software for automated electron tomography. For the TEM measurements, a drop of the nanoparticle solution was dispensed onto a 3 mm carbon-coated copper grid, and excessive solution was removed by an absorbent paper. Then the sample was dried under vacuum at room temperature. An energy dispersive X-ray spectroscopy (EDX) analyzer attached to the TEM operated in the STEM mode was used to analyze the chemical compositions of the synthesized nanoparticles. UV-visible spectra of Ag, core-shell Pt@Ag, core-shell-shell Pt@Ag@Ag-Pd and CBS Pt-Pd colloidal solutions in toluene were collected on a Hitachi U-3900 spectrophotometer. Powder X-ray diffraction (XRD) patterns were recorded on a Bruker D8 diffractometer, using Cu Kα radiation (λ = 0.154056 nm). The content of Ag in core-shell-shell Pt@Pd@Ag-Pd nanoparticles after NaCl treatment was determined using inductively coupled plasma-optical emission spectrometry (ICP-OES) technique on a Perkin Elmer 6300 spectrograph.

### Electrochemical measurements

Electrochemical measurements were carried out in a standard three-electrode cell, which was connected to a Bio-logic VMP3 (with EC-lab software version 9.56) potentiostat. A leak-free Ag/AgCl (saturated with KCl) electrode was used as the reference. The counter electrode is a platinum mesh (1 × 1 cm^2^) attached to a platinum wire.

For the loading of the catalyst on Vulcan XC-72 carbon support, a calculated amount of carbon powder was added to the toluene solution of Pt seed and CBS Pt-Pd colloidal solutions in toluene, respectively. After vigorously stirring the mixtures for 6 h, the Pt/C and CBS Pt-Pd/C (20 wt% Pt on carbon support) were collected by centrifugation and washed thrice with ethanol, followed by drying at room temperature in vacuum.

The working electrode was a thin layer of Nafion-impregnated catalyst cast on a vitreous carbon disk. This electrode was prepared by ultrasonically dispersing 5 mg of the nanoparticles/C in 1 mL of ethanol containing 0.05 mL of Nafion solution. A calculated volume of the ink was dispensed onto the 5 mm glassy carbon disk electrode to produce a nominal catalyst loading of 25.5 μg cm^−2^ (Pt basis). The carbon electrode was then dried in a stream of warm air at 70 °C for 1 h.

Electrochemical CO stripping voltammograms used to determine the electrochemically active surface areas (ECSAs) of the catalysts were obtained by the oxidation of pre-adsorbed CO (CO_ad_) in 0.1 M HClO_4_ at a scan rate of 50 mV s^−1^. CO was introduced into 0.1 M HClO_4_ for 20 min to allow for complete adsorption of CO onto the catalyst. During this process, the working electrode was maintained at 0.15 V. Excessive CO in the electrolyte was then purged out using N_2_ with high purity for 20 min. The amount of CO_ad_ was measured by integration of the CO_ad_ stripping peak, and the specific ECSA was calculated based on the following:


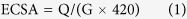


where Q is the charge of CO desorption-electrooxidation in microcoulomb (μC), which is calculated by dividing the scan rate with the integral area of CO desorption peak. G represents the total amount of Pt (μg) on the electrode, and the number (420) is the charge (μC cm^−2^) required to oxidize a monolayer of CO on the catalyst.

The performance of Pt seeds and CBS Pt-Pd nanoparticles in room-temperature MOR was measured by cyclic voltammetry. For these measurements the potential window of 0 V to 1 V was scanned at 20 mV s^−1^ until a stable response was obtained. The electrolyte was methanol (1 M) in perchloric acid (0.1 M).

The performance of carbon-supported Pt seeds and CBS Pt-Pd nanoparticles in room temperature ORR was evaluated in 0.1 M HClO_4_ electrolyte solution using a glass carbon rotating disk electrode (RDE) at a rotation rate of 1600 rpm. Negative-going linear sweep voltammograms were recorded from 1.0 to 0 V at 10 mV s^−1^ at room temperature in the presence of bubbling ultra-pure oxygen to maintain a saturated oxygen atmosphere near the working electrode. In addition, the solution with different concentration of methanol from 0.1 to 1 M in 0.1 M HClO_4_ was used for testing the methanol tolerant property of Pt seeds and CBS Pt-Pd nanoparticles. The current density was normalized by the geometric area of RDE (0.196 cm^2^) to obtain the specific activities.

## Additional Information

**How to cite this article**: Chen, D. *et al.* Cage-bell Pt-Pd nanostructures with enhanced catalytic properties and superior methanol tolerance for oxygen reduction reaction. *Sci. Rep.* **6**, 24600; doi: 10.1038/srep24600 (2016).

## Supplementary Material

Supplementary Information
