# Supplementary material for: Cage-bell Pt-Pd nanostructures with enhanced catalytic properties and superior methanol tolerance for oxygen reduction reaction
Source: Sci Rep. 2016 Apr 15;6:24600. doi: 10.1038/srep24600 (PMC4832323; doi:10.1038/srep24600)
Supplement: Supplementary Information [file srep24600-s1.doc]

Supplementary Information

**Cage-bell Pt-Pd nanoparticles with enhanced catalytic properties and superior methanol tolerance for oxygen reduction reaction**

Dong Chen^1,2^, Feng Ye^1^, Hui Liu^1,3^ & Jun Yang^1,3^,*

^1^State Key Laboratory of Multiphase Complex Systems, Institute of Process Engineering, Chinese Academy of Sciences, Beijing 100190, China. Fax: 86-10-8254 4915; Tel: 86-10-8254 4915; E-mail: [jyang@ipe.ac.cn](mailto:jyang@mail.ipe.ac.cn)

^2^University of Chinese Academy of Sciences, No. 19A Yuquan Road, Beijing 100049, China

^3^Center for Mesoscience, Institute of Process Engineering, Chinese Academy of Sciences, Beijing, 100190, China

Financial support from the National Natural Science Foundation of China (No.: 21173226, 21376247, 21476246, 21506225, 21573240), and Center for Mesoscience, Institute of Process Engineering, Chinese Academy of Sciences (COM2015A001) is gratefully acknowledged.

**Figure S1 │ X-ray diffraction (XRD) characterizations.** XRD patterns of Pt seed particles (a), Ag nanoparticles (b), Pd nanoparticles synthesized in oleylamine (c), core-shell Pt@Ag nanoparticles synthesized by seed-mediated growth (d), core-shell-shell Pt@Ag@Ag-Pd nanoparticles synthesized by galvanic replacement reaction between Ag shell of core-shell Pt@Ag nanoparticles and Pd^2+^ precursors in oleylamine (e), and cage-bell structured Pt-Pd nanoparticles (f).

**Figure S2 │ Pt seed particles.** TEM image (a) and HRTEM image (b) of the as-prepared Pt seed particles with an average diameter of 5.9 nm.

**Figure S3 │ UV-visible characterizations.** UV-Visible spectra of Ag colloidal solution by oleylamine reduction of AgNO_3_ (a), core-shell Pt@Ag nanoparticles (b), core-shell-shell Pt@Ag@Ag-Pd nanoparticles (c), and cage-bell structured Pt-Pd nanoparticles (d).

**Figure S4 │ CO stripping tests for Pt seeds and CBS Pt-Pd nanoparticles.** Cyclic voltammograms for CO stripping on Pt seed nanoparticles (a) and cage-bell Pt-Pd nanoparticles (b) in 0.1 M HClO_4_ at scan rate of 50 mV s^-1^. Black line: 1^st^ scan; red line: 2^nd^ scan.

**Figure S5 │ Durability of Pt seeds and CBS Pt-Pd nanoparticles for ORR.** Chronoamperograms at 0.55 V of Pt seeds in O_2_ saturated 0.1 M HClO_4_ electrolyte without methanol and cage-bell Pt-Pd catalysts in equal electrolyte but with 0.5 M methanol at a rotating rate of 1600 rpm.
